# Relationship Between Thyroid Function Tests and Birth Parameters at 41-Week-And-Above Pregnancies: A Prospective Cohort Study

**DOI:** 10.3390/diagnostics15050641

**Published:** 2025-03-06

**Authors:** Mustafa Can Sivas, Karolin Ohanoglu Cetinel, Ipek Emine Geyikoglu

**Affiliations:** Department of Obstetrics and Gynecology, Republic of Türkiye Ministry of Health, Basaksehir Cam and Sakura City Hospital, Istanbul 34480, Türkiye; karolinohanoglu@gmail.com (K.O.C.); ipek.geyikoglu1@gmail.com (I.E.G.)

**Keywords:** fetal distress, late-term pregnancy, thyrotropin, thyroxine, weights of newborns

## Abstract

**Background:** In the literature, there is no study investigating the relationship between thyroid hormones in pregnancies at 41 weeks and above and the birth timing, labor duration, frequency of fetal distress, premature rupture of membranes (PROM), and maternal hemogram values. **Methods:** A total of 68 nulliparous pregnant women who were admitted to Basaksehir Cam and Sakura City Hospital with indications of delivery between August 2023 and January 2024, between the ages of 20 and 38 and with no comorbidities, were included in the study. Pregnant women with ≥41 weeks of gestation were classified as the late-term pregnancy group (*n* = 37), and those between 37 and 38 weeks were classified as the control group (*n* = 31). The thyrotropin (TSH), free thyroxine (FT4), and hemoglobin levels and relevant parameters were evaluated. **Results:** The FT4 values of pregnant women diagnosed with fetal distress in the entire population were observed to be statistically significantly lower (*p* < 0.05). A statistically significant negative linear relationship was detected between the FT4 values of the entire population and the weights of newborns (*p* < 0.05). It was determined that, as the FT4 values decreased, the newborn weights increased. There was no statistically significant difference between the two groups in terms of the TSH/FT4 values, birth types, labor duration, or postpartum Hb/Htc decrease (*p* > 0.05). No statistically significant relationship was found between the TSH/FT4 values of the entire population and the diagnosis of PROM, labor duration, or Hb/Htc decrease amount (*p* > 0.05). **Conclusions:** TSH/FT4 levels may be important in the mature and late–mature periods of pregnancy. There may be an association between the FT4 levels and the fetal distress risk, type of birth, and newborn weight.

## 1. Introduction

Thyroid hormones have significant effects on human physiology. Any decrease or increase in hormone levels may cause changes in cellular metabolism. Numerous hormones play a role, from the hypothalamic–pituitary axis to the thyroid gland and beyond. Free thyroxine (FT4) and free triiodothyronine (FT3) are the active thyroid hormones secreted by the thyroid gland. Since most of FT3 is formed by the deiodination of FT4 in the peripheral tissue, the FT4 level is often used in analyses. The secretion of these hormones is controlled by thyrotropin (TSH). While disorders related to the functioning of the thyroid gland are referred to as primary thyroid diseases, secretion changes originating from the hypothalamic–pituitary axis are referred to as secondary thyroid diseases. The TSH level is one of the main guides in distinguishing primary and secondary thyroid diseases. Therefore, TSH and FT4 are the hormones most commonly used to assess thyroid function [1,2,3]. In the case of high TSH levels and decreased FT4 levels, primary hypothyroidism is mentioned [4]. In the case of low TSH values and increased FT4 levels, primary hyperthyroidism is mentioned [5]. Hypothyroidism may lead to weight gain, tiredness, hair loss, a depressed mood, and cold intolerance [6,7]. However, palpitations, weight loss, tremors, and hyperarousal are symptoms in hyperthyroidism [8].

Thyroid hormones are especially important in pregnancy. The developing fetus and ongoing pregnancy are affected by hormone levels. A pregnancy with untreated hypothyroidism can lead to children who have a low intelligence quotient (IQ) [9,10]. Thyroid hormones play a decisive role in neuronal migration, cortex development, synapse formation, synapse numbers, and cellular apoptosis activity in this process [11,12]. In the studies carried out, it has been shown that the thyroid hormones have effects on the progress of pregnancy and even the timing of birth. The preterm labor rates are higher in pregnant women with hypothyroidism or isolated hypothyroxinemia. In isolated hypothyroxinemia, while TSH is within the normal limits, FT4 is below the normal value [13,14].

While there are studies examining the relationship between thyroid function tests and preterm birth, there are few studies in the literature examining the relationship between thyroid function tests and the late stages of mature pregnancy. Studies examining the relationship between thyroid function tests and women with a pregnancy over 41 weeks are very limited. In the literature, we determined that there is no study investigating the relationship between thyroid hormones in pregnancies of 41 weeks and above and the birth timing, labor duration, frequency of fetal distress, premature rupture of membranes (PROM), and maternal hemogram values. There may be a relationship between these birth parameters and thyroid hormones. In such a case, factors that increase mortality and morbidity, such as fetal distress, peripartum anemia, prolonged labor, etc., can be predicted. Preventive treatments and follow-ups can be planned.

In this context, the aim of this study was to evaluate the relationship between thyroid hormones and the dynamics and morbidities during the birth process by prospectively analyzing the thyroid function test values of pregnant women who were hospitalized and delivered at the 41st week and later.

## 2. Materials and Methods

This study was planned and completed as a prospective cohort study. This study was performed in line with the principles of the Declaration of Helsinki. Informed consent in accordance with ethical requirements was obtained from all participants. Approval was granted by the Ethics Committee of the Basaksehir Cam and Sakura City Hospital, Istanbul, Türkiye (date: 12 July 2023, protocol no.: 310).

### 2.1. Design of the Study

Pregnant women who were admitted to the Basaksehir Cam and Sakura City Hospital with the indication of delivery between August 2023 and January 2024, who were nulliparous, between the ages of 20 and 38, and who did not have any comorbidities, were included in the study. While determining the gestational week, calculations were performed according to the gestational week obtained from the crown–rump length (CRL), which was measured at the ultrasound examination performed between 8 and 14 weeks of pregnancy, and the final gestational week was obtained. The study continued with a total of 70 evaluable patients who met the standardization criteria. Two patients in the control group were excluded from the study due to blood results showing clots. Two groups were created:

The late-term pregnancy group ([LTP], *n* = 37): pregnant women with ≥41 weeks of gestation were included;
The control group ([C], *n* = 31): pregnant women between 37 and 38 weeks were included.

Thyroid function tests (TSH, FT4) and hemograms were performed in all pregnant women during their admission to the delivery room. The labor duration, fetal distress rates, frequency of PROM, presence of meconium fluid, newborn weights, and 24th-hour postpartum hemoglobin/hematocrit (Hb/Htc) values of the same pregnant women were monitored. For the standardization of the labor duration, the 4 cm dilation was taken as the basis for the beginning of labor. In pregnant women with effective uterine contraction, the time from 4 cm dilation to birth was calculated. Pregnant women admitted to the delivery room with dilation of >4 cm were included in the parameter evaluations but were not included in the evaluation of the labor duration. The diagnosis of fetal distress and the decision to perform a cesarean section were based on repeated late decelerations and a lack of response to hydration, oxygen, turning to the left side, and mechanical stimulation, or no return to baseline fetal heart activity (fetal heart < 110 beats per minute) [15]. The difference between the Hb/Htc values at the time of admission to the delivery room and the Hb/Htc values at the 24th hour postpartum was calculated. After the relevant parameter evaluations were completed for all patients, the statistical significance level was checked to determine whether there was a difference between the groups.

### 2.2. Inclusion and Exclusion Criteria

Pregnant women at 41 weeks and above and pregnant women between 37 and 38 weeks were included in the study. Pregnant women who were hospitalized with indications for labor and who were about to give birth for the first time, who had a normal vaginal birth without intervention as a result of their follow-up, were included in the study. In order to exclude factors that could affect the thyroid values, pregnant women using additional medications other than iron preparations and/or multivitamins, and pregnant women with any additional diseases, such as diabetes, hypertension, a diagnosed thyroid disease, autoimmune diseases, etc., were not included in the study.

### 2.3. Statistical Analysis

When determining the sample size, calculations were performed in accordance with the protocol using the GPower3.1 software (version 3.1.9.7, Heinrich Heine University Düsseldorf, Düsseldorf, Germany) based on an alpha error probability of 0.05 and a power value of 0.80. For the statistical analysis, SPSS Version 22.00 (IBM Corporation, Armonk, NY, USA) was used. To evaluate the distribution of the data, the Kolmogorov–Smirnov test was used. Variables complying with a normal distribution were given as the mean ± standard deviation, and an independent-samples *t*-test was used in comparisons between two independent groups. Variables that did not comply with a normal distribution were given as median (minimum–maximum) values, and the Mann–Whitney U test was used in comparisons between two independent groups. Categorical variables were given as frequency (*n*) and percentage (%) values, and the Pearson chi-squared test was used in the comparisons. Relationships between the variables were examined with Pearson and Spearman correlation coefficients. A *p*-value of 0.05 was taken as the threshold level for statistical significance.

## 3. Results

After applying the inclusion/exclusion criteria, this study continued with a total of 70 evaluable patients. Two patients in the control group were excluded from the study due to blood results showing clots. The study was completed with a total of 68 pregnant women: 37 in the late-term pregnancy group and 31 in the control group.

### 3.1. The Relationship Between Thyroid Hormones and the Parameters of the Birth Process

There was no statistically significant difference between the two groups in terms of the TSH and FT4 values (*p* > 0.05) (Table 1).

Twenty pregnant women in the LTP group and 12 pregnant women in the C group delivered by cesarean section. Considering pregnant women who gave birth by cesarean section, there was no statistically significant difference between the LTP and C groups in terms of the TSH and FT4 values (*p* > 0.05) (Table 2). When the entire population was divided into two, namely those who delivered by cesarean section or non-assisted vaginal delivery (NVD), no statistically significant difference was observed between the two groups in terms of the TSH and FT4 values (*p* > 0.05) (Table 3).

A statistically significant relationship was found between the FT4 values of the entire population and the diagnosis of fetal distress (*p* < 0.05). The FT4 values of pregnant women diagnosed with fetal distress were observed to be lower. No statistically significant relationship was found between the TSH values and a fetal distress diagnosis (*p* > 0.05); although no significant relationship was found, the TSH values were observed to be higher in patients diagnosed with fetal distress (Table 4).

**Figure 1:**
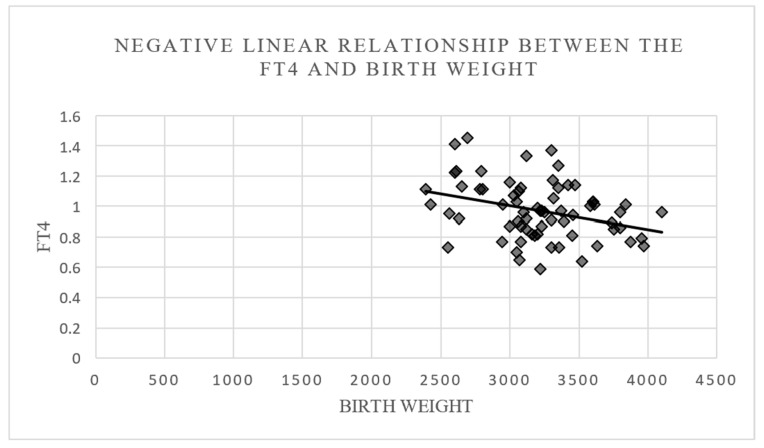
Negative linear relationship between the FT4 and birth weight.

No statistically significant relationship was found between the TSH/FT4 values of the entire population and the diagnosis of PROM, the labor duration, or the amount of Hb/Htc decrease (*p* > 0.05). A statistically significant negative linear relationship was detected between the FT4 values of the entire population and the weights of newborns (*p* < 0.05). It was determined that, as the FT4 values decreased, the newborn weights increased (Figure 1). No statistically significant relationship was found between the TSH values of the entire population and the weights of the newborns (*p* > 0.05) (Table 5).

### 3.2. The Relationship Between the Gestational Week and Parameters of the Birth Process

There was a statistically significant difference between the two groups in the diagnosis of PROM. It was determined that the rate of diagnosis of PROM was lower in participants who gave birth at ≥41 weeks than in participants who gave birth at 37–38 weeks (C, LTP; 51.6%, 2.7%, *p* < 0.001). There was a statistically significant difference between the two groups in terms of the newborn weight. The weight of newborns with a birth time of ≥41 weeks was observed to be higher compared to those with a birth time of 37–38 weeks (median gr; C, LTP; 3026, 3366, *p* < 0.001). No statistically significant relationship was observed between the birth time and birth type among the entire population (*p* = 0.207). There was no statistically significant difference between the two groups in terms of a fetal distress diagnosis, the labor duration, or a postpartum Hb/Htc decrease (*p* > 0.05). Considering the pregnant women who had a normal vaginal birth, similarly, no statistically significant difference was observed between the two groups in the Hb/Htc decrease amount (median Hb g/dL; C, LTP; 1.01, 1.15, *p* = 0.586) (median Htc %; C, LTP; 3.94, 3.83, *p* = 0.914).

## 4. Discussion

In this study, it was determined that thyroid hormones do not play a primary role in the progression of birth time to the late mature period. No relationship was found between thyroid hormones and the type of birth. However, when the indications for a cesarean section were limited to a diagnosis of fetal distress, lower FT4 values were associated with more diagnoses of fetal distress. Although no significant relationship was found between the diagnosis of fetal distress and the TSH value, the TSH values were observed to be higher in fetal distress patients. It is understood from the higher TSH and lower FT4 values that the thyroid glands of pregnant women diagnosed with fetal distress work relatively less effectively than those of the whole population. Lower FT4 values were associated with higher newborn weights, and a negative linear relationship was found. No relationship was found between thyroid hormones and being diagnosed with PROM, the labor duration, or the postpartum bleeding amount.

In evaluations based on the week of birth, it was found that the frequency of PROM was higher between 37 and 38 weeks. It was determined that babies born at 41 weeks and later had higher newborn weights, as expected. It was determined that giving birth in the late mature period had no effect on being diagnosed with fetal distress, the labor duration, or the postpartum Hb/Htc decrease amount, compared to giving birth at 37–38 weeks. Considering the entire population, it was determined that the week of birth does not play a determining role in the type of birth.

Thyroid hormones have a regulatory role in body development and metabolism. Similarly, thyroid hormones are effective in placental development and function and fetal development [16,17,18]. In the study conducted by Barjaktarovic et al. [16], high FT4 values in the third trimester were associated with an increase in the umbilical artery pulsatility index and an increased risk of notch in the uterine artery. On the other hand, no relationship was found between the TSH value and the same parameters, which are related to placentation. It was stated that the effect of FT4 on cytokines and growth factors such as VEGF-A, EGF, IL-10, and TNF-α may have played a role in these results [16]. In studies conducted in the literature, thyroid hormones have been shown to have effects on the onset of labor. The expression of neuropeptides involved in the onset of labor was affected by the thyroid hormone levels [19]. Lee et al. determined that there was a two-fold increased risk of premature birth in pregnant women with TSH levels >4 mIU/L [20]. Rahmati et al. stated in their study’s conclusions that “regardless of the TPOAb status or iodine insufficiency, the risk of preterm labor is increased in pregnant women with a TSH value of >3.92 mIU/L” [21]. Additionally, Yang et al. found that the risk of preterm delivery may increase also in cases of subclinical hypothyroidism [22]. In the meta-analysis conducted by the “Consortium on Thyroid and Pregnancy-Study Group on Preterm Births”, the preterm labor rates were higher in pregnant women with subclinical hypothyroidism or isolated hypothyroxinemia [13]. It has been observed that low-dose levothyroxine treatment may reduce the rates of miscarriage or premature birth in pregnant women with subclinical hypothyroidism [21,23,24,25]. In our study, no relationship was found between thyroid hormones and the time of onset of labor or the labor duration in pregnancies at 37 weeks and above. The lack of relationship between thyroid hormones and the labor duration suggests that thyroid hormones do not have a determining role in the release of the oxytocin hormone; however, since the blood oxytocin levels were not measured in this study, it would not be appropriate to make further comments. In addition, local cervical prostaglandin release, which may affect the labor duration, is another major mechanism that needs to be investigated.

In a few studies in the literature, thyroid function values have been investigated in the term, late-term, or post-term period. In one of these studies, it was stated that the cesarean section rates were higher in pregnant women with hypothyroidism. However, apart from hypothyroidism, the reason for these high cesarean section rates is as follows: it has been stated that there may be additional diseases, such as hypertensive diseases, preterm labor, or gestational diabetes [26]. In our study, pregnant women without comorbidities were included in the study population. There was no significant relationship between the cesarean section rates and thyroid hormone values. However, it was determined that the FT4 values of pregnant women diagnosed with fetal distress were significantly lower. While significance was achieved in fetal distress, we believe that the reason that no significance was found between the cesarean section rates and low FT4 values is related to the number of participants. In studies involving a larger patient population without comorbidities, a higher number of fetal distress diagnoses associated with low FT4 values may exhibit statistical significance regarding the cesarean section rates.

In a meta-analysis by Derakhshan et al., it has been stated that, in the presence of hyperthyroidism or hypothyroidism in pregnant women, the newborn weights decrease and the risk of small for gestational age (SGA) infants increases. In the case of isolated hypothyroxinemia, it has been determined that the risk of SGA decreases and the frequency of higher birth weights increases [27]. In our study, consistent with the results of Derakhshan et al., we observed that the babies of pregnant women with lower FT4 values had higher birth weights. Additionally, for the first time in the literature, we determined that there was a negative linear relationship between the FT4 values and newborn weights. Since maternal FT4 can cross the placenta, the maternal FT4 levels are effective in fetal concentration. In this respect, it is thought that the change in birth weight may be a result of the known direct effect of FT4 on weight. However, the low fetal birth weight in hypothyroidism suggests that there are mechanisms other than this physiological effect. As Derakhshan et al. also stated, isolated low FT4 levels may be a parameter indicating uteroplacental function [27]. There are not many studies in the literature on the pathophysiology of this relationship, and detailed studies are needed on this subject. Sert et al., who investigated the relationship between the TSH level and birth weight, determined that the TSH level was not related to the birth weight [28]. Similarly, our study results indicated that TSH was not effective regarding the birth weight. In the literature, while a significant relationship has been found between FT4 and fetal brain development, placentation parameters, or pregnancy outcomes regarding preeclampsia, the same relationship was not found with TSH [11,12,16]. When all of these results are evaluated together, it is understood that FT4 may be the main determinative thyroid hormone when investigating the relationship between parameters related to pregnancy and thyroid hormones. However, Monen et al. determined in their study, which prospectively examined the thyroid hormone values of 1051 pregnant women in each trimester, that high TSH was associated with meconium-containing amniotic fluid in pregnancies at 41 weeks and above. In this study, it was determined that, in cases of high TSH, the incidence of meconium-containing amniotic fluid increased significantly after 41 weeks. Additionally, it was determined that the TSH values of these patients were higher in all trimesters [29]. In our study, since there were no pregnant women with meconium-containing amniotic fluid at the time of birth, further evaluation could not be performed regarding this issue.

This is the first study in the literature to investigate the relationship between thyroid hormones in pregnancies at 41 weeks and above and fetal distress, PROM, the labor duration, the timing of birth, and the postpartum maternal Hb/Htc decrease amount. Additionally, this is the first study to show a negative linear relationship between FT4 and the birth weight in a standardized group model without comorbidities. This study, by comparing the mature and late mature periods of pregnancy, contributes to the literature on how the gestational age affects birth parameters.

### Limitations of the Study

In this study, while participants with known thyroid diseases and other comorbidities were excluded, additional factors such as the nutritional status, iodine intake, and socioeconomic background were not evaluated. Physiologically, in the first trimester, T4 production increases with the effect of human chorionic gonadotropin hormone. As the synthesis of thyroid hormones increases, the need for iodine increases. From the second trimester onwards, the effect of human chorionic gonadotropin hormone decreases and the TSH level increases to meet the maternal metabolic requirements. In the third trimester, thyroid function becomes more stable, while the iodine requirement becomes more prominent [28]. The nutritional status, iodine intake, and socioeconomic background should be taken into consideration in new studies because they could influence physiological changes in thyroid function across gestational ages. The second limitation is the number of participants. The sample size in this study is a factor that may limit the generalizability of the results to broader populations. Studies with a larger patient population are needed, especially to demonstrate the relationship between low FT4 hormone levels and increased cesarean section rates. Although no difference was observed in the thyroid hormone values between those who gave birth at 37–38 weeks and those who gave birth at 41 weeks and above, it cannot be determined, based on the data of this study, how hyperthyroidism or hypothyroidism affect the birth timing of pregnancies over 37 weeks. There is a need for new studies that classify patients as euthyroid, hyperthyroid, and hypothyroid and investigate the late mature period. The power of such studies will be increased if the thyroid levels at the beginning of the pregnancy and the levels in the first and second trimesters are also taken into account, because the thyroid hormones show dynamic changes between trimesters and affect the mother and fetus. Another limitation is that a randomization method was not applied in this study. Patients who met the inclusion/exclusion criteria were included in the study prospectively according to their order of admission to the hospital. A randomized patient selection method in studies with larger patient populations will increase the power of such studies by eliminating unknown factors that may affect the study results. Moreover, in new studies, when making comparisons between groups, taking into account the body mass index values and thyroid peroxidase antibody positivity of the patients may provide a more precise evaluation of thyroid function.

## 5. Conclusions

Thyroid hormone levels may be important in the mature and late–mature periods of pregnancy. There may be an association between FT4 hormone levels and the risk of fetal distress, type of birth, and newborn weight.

## Figures and Tables

**Table 1:** Comparison of TSH and FT4 values between the groups.

	Group	*n*	Mean ± SD	*p* Value
TSH	C	31	2.39 ± 1.052	0.703
	LTP	37	2.29 ± 0.909	
FT4	C	31	1.01 ± 0.204	0.159
	LTP	37	0.94 ± 0.170	

*p* < 0.05, independent-sample *t*-test was used. C, control; LTP, late-term pregnancy. TSH, uIU/mL; FT4, ng/dL.

**Table 2:** Analysis of TSH and T4 values of pregnant women who had a cesarean section according to their gestational week.

	Group	*n*	Mean ± SD	*p* Value
TSH	C	12	2.69 ± 1.361	0.626
	LTP	20	2.48 ± 1.040	
FT4	C	12	0.99 ± 0.235	0.241
	LTP	20	0.90 ± 0.171	

*p* < 0.05, independent-sample *t*-test was used. C, control; LTP, late-term pregnancy. TSH, uIU/mL; FT4, ng/dL.

**Table 3:** Analysis of TSH and FT4 values according to the type of birth.

	Type of Birth	*n*	Mean ± SD/Median (Min–Max)	*p* Value
TSH	NVD	36	2.07 (1–4)	0.131 **
	C/S	32	2.37 (1–5)	
FT4	NVD	36	1.01 ± 0.174	0.102 *
	C/S	32	0.93 ± 0.198	

*p* < 0.05, * independent-sample *t*-test was used, ** Mann–Whitney U Test was used. NVD, non-assisted vaginal delivery; C/S, cesarean section. TSH, uIU/mL; FT4, ng/dL.

**Table 4:** The relationship between the TSH and FT4 values and a fetal distress diagnosis.

	Fetal Distress	*n*	Mean ± SD	*p* Value
TSH	-	52	2.29 ± 0.976	0.473
	+	16	2.49 ± 0.965	
FT4	-	52	1 ± 0.188	0.019
	+	16	0.88 ± 0.160	

*p* < 0.05, independent-sample *t*-test was used. TSH, uIU/mL; FT4, ng/dL.

**Table 5:** The relationship between the TSH and FT4 values and birth weight.

		TSH	FT4
Birth Weight	r	−0.213	−0.274
	*p*	0.082	0.024

*p* < 0.05, Pearson correlation analysis was used.

## Data Availability

The datasets generated and/or analyzed during the current study are not publicly available due to local ethical and legal requirements but are available from the corresponding author on reasonable request.

## Abbreviations

The following abbreviations are used in this manuscript:

IQ	Intelligence quotient
TSH	Thyrotropin
FT4	Free thyroxine
LTP	Late-term pregnancy
C	Control
Hb	Hemoglobin
Htc	Hematocrit
NVD	Non-assisted vaginal delivery
SGA	Small for gestational age
